# Role of the circulating milieu in age-related arterial dysfunction: a novel ex vivo approach

**DOI:** 10.1152/ajpheart.00014.2024

**Published:** 2024-03-22

**Authors:** Sophia A. Mahoney, Nicholas S. VanDongen, Nathan T. Greenberg, Ravinandan Venkatasubramanian, Matthew J. Rossman, Michael E. Widlansky, Vienna E. Brunt, Yara Bernaldo de Quirós, Douglas R. Seals, Zachary S. Clayton

**Affiliations:** ^1^Department of Integrative Physiology, University of Colorado Boulder, Boulder, Colorado, United States; ^2^Department of Medicine and Pharmacology, Medical College of Wisconsin, Milwaukee, Wisconsin, United States; ^3^Department of Medicine, University of Colorado Anschutz Medical Campus, Aurora, Colorado, United States; ^4^Institute of Animal Health and Food Safety, Universidad de Las Palmas de Gran Canaria, Canary Islands, Spain

**Keywords:** aging, arterial stiffness, cardiovascular disease, circulating milieu, endothelial function

## Abstract

The circulating milieu, bioactive molecules in the bloodstream, is altered with aging and interfaces constantly with the vasculature. This anatomic juxtaposition suggests that circulating factors may actively modulate arterial function. Here, we developed a novel, translational experimental model that allows for direct interrogation of the influence of the circulating milieu on age-related arterial dysfunction (aortic stiffening and endothelial dysfunction). To do so, we exposed young and old mouse arteries to serum from young and old mice and young and midlife/older (ML/O) adult humans. We found that old mouse and ML/O adult human, but not young, serum stiffened young mouse aortic rings, assessed via elastic modulus (mouse and human serum, *P* = 0.003 vs. young serum control), and impaired carotid artery endothelial function, assessed by endothelium-dependent dilation (EDD) (mouse serum, *P* < 0.001; human serum, *P* = 0.006 vs. young serum control). Furthermore, young mouse and human, but not old, serum reduced aortic elastic modulus (mouse serum, *P* = 0.009; human serum, *P* < 0.001 vs. old/MLO serum control) and improved EDD (mouse and human serum, *P* = 0.015 vs. old/MLO serum control) in old arteries. In human serum-exposed arteries, in vivo arterial function assessed in the human donors correlated with circulating milieu-modulated arterial function in young mouse arteries (aortic stiffness, *r* = 0.634, *P* = 0.005; endothelial function, *r* = 0.609, *P* = 0.004) and old mouse arteries (aortic stiffness, *r* = 0.664, *P* = 0.001; endothelial function, *r* = 0.637, *P* = 0.003). This study establishes novel experimental approaches for directly assessing the effects of the circulating milieu on arterial function and implicates changes in the circulating milieu as a mechanism of in vivo arterial aging.

**NEW & NOTEWORTHY** Changes in the circulating milieu with advancing age may be a mechanism underlying age-related arterial dysfunction. Ex vivo exposure of young mouse arteries to the circulating milieu from old mice or midlife/older adults impairs arterial function whereas exposure of old mouse arteries to the circulating milieu from young mice or young adults improves arterial function. These findings establish that the circulating milieu directly influences arterial function with aging.

## Graphical abstract

**Figure:**
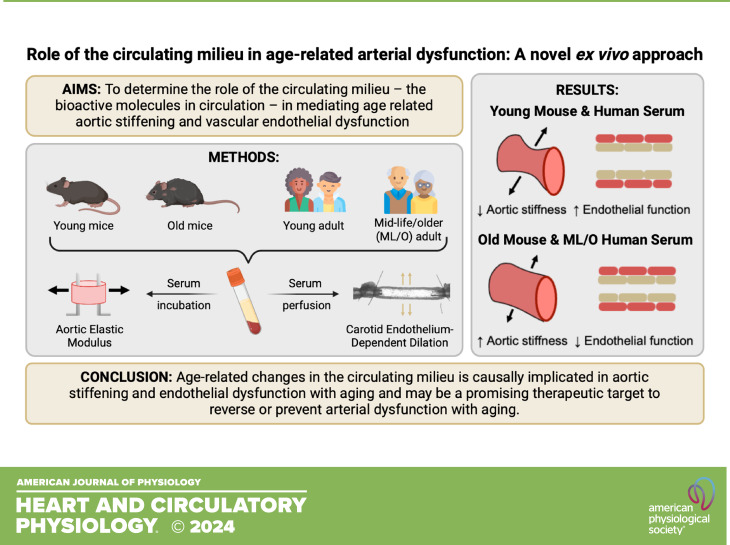

## INTRODUCTION

Advancing age is the primary nonmodifiable risk factor for the development of cardiovascular diseases (CVDs), which remain the leading cause of mortality worldwide (1, 2). The key pathophysiological antecedent to the development of clinical CVD with aging is arterial dysfunction (3). The two most clinically important manifestations of age-related arterial dysfunction are large elastic artery (e.g., aortic) stiffening and impaired vascular endothelial function (3). Currently, the underlying mechanisms mediating age-related arterial dysfunction are incompletely understood.

The circulating milieu, the bioactive factors in the bloodstream, is altered with advancing age, shifting to a more inflammatory and prooxidant phenotype in midlife/older (ML/O) adults compared with young adults (4). These age-related changes in the circulating milieu have adverse effects on physiological function in selective cell types and tissues (2, 4–6). Vascular cells and arteries may be particularly vulnerable to age-related alterations in the circulating milieu given the direct, constant interaction between arteries and the bloodstream (7). Current experimental approaches used to study the circulating milieu (e.g., heterochronic parabiosis and heterochronic blood exchange) suggest that there may be a relationship between changes in the circulating milieu with aging and arterial dysfunction (8). However, because of potential in vivo systemic influences on arterial function (e.g., immune responses), these experimental approaches are unable to establish direct evidence of the causal effect of the circulating milieu on arterial function (9). Moreover, causal evidence to date is limited to the effects of the circulating milieu from preclinical animal models on cellular and tissue function (10); as such, it is unknown if the circulating milieu from humans induces age-related effects on function in intact arteries. Given the incomplete understanding of the role of the circulating milieu in mediating age-related arterial dysfunction, it is biomedically important to develop new strategies that allow for direct interrogation of this possible mechanism.

In this study, we aimed to establish a novel, translational model in which we could directly interrogate the influence of the circulating milieu of mice and humans in age-related arterial function. To do so, we developed ex vivo serum exposure models in which isolated arteries from young and old mice were exposed to serum obtained from either young or old mice. We first established that arteries exposed to serum from age-matched mice had arterial function that was reflective of their age. We then studied the age-related effects of the circulating milieu by exposing arteries to serum from mice of different age. We found that this heterochronic serum exposure induced a bidirectional transfer of age-related arterial function in which young arteries had lower function following exposure to old serum and old arteries had improved function following exposure to young serum. We next translated our findings to human participants and found that young and ML/O adult serum could transduce similar phenotypes in mouse arteries as previously demonstrated with mouse serum (i.e., young mouse arteries had impaired function following exposure to ML/O adult human serum and old mouse arteries had improved function following exposure to young adult human serum). We then showed that circulating milieu-mediated changes in arterial function were related to the participants’ in vivo arterial function, providing initial insight into the putative role of the circulating milieu in mediating arterial function in vivo in humans. The innovative methodological approaches established in this study identify the circulating milieu as an important contributor to age-related arterial dysfunction and a promising therapeutic target for improving arterial function with aging.

## MATERIALS AND METHODS

### Animal Ethical Approval

All mouse studies and procedures were reviewed and approved by the Institutional Animal Care and Use Committee at the University of Colorado (CU) Boulder (Protocol No. 2618). All procedures adhered to the guidelines set forth by the National Institutes of Health’s *Guide for the Care and Use of Laboratory Animals* (11).

### Animal Studies

Young (3–5 mo) and old (25 mo) male and female, intervention naïve C57BL/6N mice obtained from the National Institute on Aging colony (maintained by Charles River, Wilmington, MA) were used in these studies. Mice were allowed to acclimate to our facilities for at least 2 wk before beginning the study. For the duration of the study, all mice were single-housed at our animal facility with a 12-h:12-h light/dark cycle and allowed ad libitum access to an irradiated, fixed, and open rodent chow (Inotiv/Envigo 7917, stored at room temperature) and drinking water (Boulder, CO; municipal tap water that underwent reverse osmosis and chlorination).

### Participants

Data were obtained from 23 healthy adults (young, 18–29 yr or ML/O, 50+ yr) previously studied by our laboratory during the 2020–2023 period. All procedures were approved by the Institutional Review Board of CU Boulder. Participants included in this study consented to perform follow-up analyses with samples collected during their visits. Participants underwent blood collection, baseline testing for casual (resting) blood pressure, aortic stiffness, and vascular endothelial function by our laboratory with well‐standardized procedures at CU Boulder. All measurements were performed after a 12-h fast from food (water permitted) and caffeine; a 24-h abstention from alcohol, physical activity, prescription medications, and a 48-h abstention from over-the-counter medications and supplements.

Serum samples, subject characteristics, and functional data from standard visits were used in this study. Participants were nonobese (body mass index < 30 kg/m^2^), nonsmokers, nondiabetic, and free of other clinical diseases as determined by medical history, physical examination, and blood chemistry. To minimize the influence of female sex hormones on our outcomes, premenopausal women underwent testing during low-hormone conditions, i.e., during the early follicular phase of their menstrual cycle (*days 1–7*) if naturally menstruating or during the placebo phase if taking hormonal contraceptives.

### Clinical Measurements

Clinical measurements were performed at the CU Boulder Clinical Translational Research Center. Body mass index and total body fat as well as resting arterial systolic and diastolic blood pressures were determined as previously described (12, 13). Leisure time physical activity was determined by the self-reported modifiable activity questionnaire (14).

Aortic stiffness was assessed using the reference standard noninvasive in vivo measurement, carotid femoral pulse wave velocity (cfPWV), by applanation tonometry with simultaneous ECG gating of the R wave to measure the time delay between the foot of the carotid and femoral arterial pressure waves, as previously described (15, 16). cfPWV was calculated as the distance between arterial sites (in m) divided by the arterial pressure wave transit time (in s) at each site, with automated software (Non-Invasive Hemodynamics Workstation; Cardiovascular Engineering Inc).

Brachial artery flow-mediated dilation (baFMD), the reference standard noninvasive approach for measuring vascular endothelial function in people, was determined using duplex ultrasonography (Toshiba, multifrequency linear-array transducer) as previously described (15, 17). baFMD is expressed as the percent change in arterial diameter from baseline after reperfusion following a 5-min forearm cuff occlusion to induce a period of forearm ischemia and reactive hyperemia.

### Experimental Design

**Figure 1.**
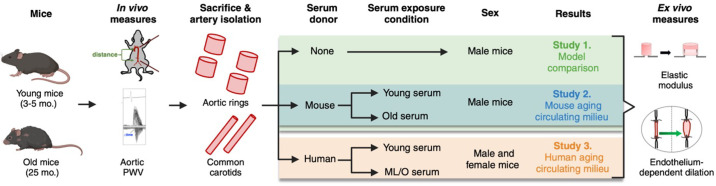
Experimental design. Young and old C57Bl6/N mice underwent in vivo assessment of aortic stiffness, via aortic pulse wave velocity (PWV), before euthanasia and isolation of the large elastic arteries (aorta and common carotid arteries). To assess the role of the circulating milieu on arterial function, isolated arteries were exposed to serum from mice or humans. Following serum exposure, elastic modulus and endothelium-dependent dilation were measured in aortas and carotid arteries, respectively. ML/O, midlife/older. Figure created with Photoshop.

To compare the serum exposure model to standard measures of age-related arterial function, we used young and old intervention-naïve mice that were recently studied by our laboratory (18) to serve as a model control for the experimental serum exposure models and accurately represent the arterial phenotypes typically observed in old C57BL/6N mice (10, 19, 20). For the validation of the models, existing data from seven young and seven old male mice were used to compare the effects of age-matched mouse serum exposure on six young and eight old male mice (i.e., young mouse arteries exposed to young mouse serum and old mouse arteries exposed to old mouse serum) (Fig. 1, green). This approach allowed us to examine age-related arterial function with and without the influence of serum and incubation time, to ultimately determine if age-matched serum influenced arterial function.

To investigate the effects of the circulating milieu on age-related arterial function, we performed serum exposure experiments with mouse arteries and serum from mice and humans of different ages. For the mouse serum exposure experiments, six young and eight old male mice were used (Fig. 1, blue). Only male mice were included in the initial studies to apply an accurate comparison of the serum exposure model above to established arterial function methodologies in which male mice have historically been used (18). After assessing the age-related effects of mouse serum exposure on arterial function, we used a similar experimental approach to assess the effects of human serum exposed to mouse arteries. For the human serum exposure experiments, five young female, five young male, five old female, and five old male mice were used and arteries from these mice were exposed to sex-matched human serum (Fig. 1, orange). Given the lack of sex differences observed, male and female data were combined in the results.

### In Vivo Aortic Stiffness

To establish that the mice used in the present study reflected typical age-related arterial dysfunction in vivo, we assessed aortic pulse wave velocity (aPWV), which is considered the noninvasive in vivo reference standard for assessing large elastic artery stiffness. aPWV was assessed 1 wk before euthanasia, as previously described (10, 18, 19). Briefly, mice were placed under light isoflurane anesthesia (1.0–2.5%) and positioned supine on a warmed heat pad. Front- and hindlimb paws were then secured to corresponding ECG electrodes. Two Doppler probes were then placed on the skin at the transverse aortic arch and the abdominal aorta. Once clear R-waves were registered, three repeated 2-s ultrasound tracings were recorded and average preejection time (time between the R-wave of the ECG to the foot of the Doppler signal) was determined for each location. To calculate aPWV, the distance between the two probes was divided by the difference between the transverse aortic arch and abdominal aorta preejection times (time_abdominal_ – time_arch_) (in cm/s).

### Ex Vivo Aortic Stiffness

To determine if the serum exposure or incubation period had effects on age-related aortic stiffness, we sought to compare the elastic modulus of aortic rings that were nonincubated (standard) or aortic rings that were cultured in age-matched serum (described in the next section). Aortic elastic modulus is a well-established ex vivo experimental approach for assessing aortic intrinsic mechanical wall stiffness with aging (10, 18, 19). Aortic elastic modulus data from standard, nonincubated aortic rings were obtained from a previous study in our laboratory (18) to use as a methodological control and asses the influence of serum exposure and incubation. For standard elastic modulus testing, aortas were promptly excised from the mice following carotid artery excision, rinsed with cold PSS, and cleared of any remnant perivascular adipose and connective tissue. To measure ex vivo aortic stiffness, two thoracic aorta samples (∼1 mm in length) were cut and used to determine intrinsic mechanical stiffness via pin myography as we have previously described (10, 18, 19). In short, aorta samples were placed in heated (37°C) baths filled with calcium-free phosphate-buffered saline (PBS). The samples were then mounted on two pins, followed by three rounds of prestretching. Once prestretching was complete, aortic ring diameter was increased until 1 mN of force was reached and incrementally increased by 5 µm every 3 min thereafter until failure. The force corresponding to each stretching interval was recorded and used to calculate stress and strain. A stress-strain curve was then generated using the following equations:

$$\text{Strain }\left(\lambda \right)=\frac{\Delta d}{d_{i}}$$

where *d* is diameter and *d_i_* is initial diameter.

$$\text{Stress }\left(t\right)=\frac{\text{ΔL}}{2(HD)}$$

where L is one-dimensional load, *H* is intima-media thickness, and *D* is vessel length.

Given that adjacent thoracic aortic sections from the same donor mice were incubated simultaneously with young versus old serum samples and subsequently underwent elastic modulus assessment at the same time, we concluded that vessel diameter and intima-media wall thickness would not be a factor that changed elastic modulus. As such, we used reference values for young mice (613 µm diameter and 39 µm thickness) and old mice (636 µm diameter and 60 µm thickness) from our laboratory. The elastic modulus of the stress-strain curve was determined as the slope of the linear regression fit to the final four points of the stress-strain curve, as previously reported by our laboratory (10).

### Circulating Milieu-Related Aortic Stiffness

**Figure 2.**
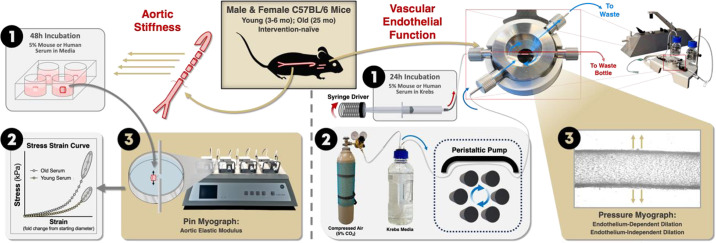
Experimental model set up. Circulating milieu-related aortic stiffness was assessed in aorta rings following *1*) 48-h incubation in mouse or human serum in media. Following serum exposure, aortic elastic modulus was *2*) measured on a pin myograph and *3*) calculated from a stress-strain curve. Circulating milieu-related vascular endothelial function was assessed in common carotid arteries following *1*) 24 h of intraluminal perfusion with mouse or human serum in Krebs media. Carotid arteries were *2*) maintained in a bath of 5% CO_2_ gas-infused Krebs media that was displaced using a peristaltic pump. Carotid arteries were heat controlled (37°C) and pressurized (50–55 mmHG; not shown). *3*) Vascular endothelial function was measured via endothelium-dependent and -independent dilation in a pressure culture myograph. Figure created with Photoshop.

Aortas were promptly excised from the mice following carotid artery excision, rinsed with cold PSS, and cleared of any remnant perivascular adipose and connective tissue. Four aortic segments from each donor animal were incubated in a 96-well plate containing mouse or human serum samples for 48 h. All serum samples were sex matched to the aorta donor animals, and paired aortic segments from each mouse donor were exposed to each condition (young or old serum) in duplicate and averaged. Serum samples from two mice were pooled to obtain sufficient material. Serum samples from humans underwent heat treatment at 56°C for 30 min to inactivate the complement cascade before culturing (21). Serum complement inhibition prevents immunological assaults on cells from foreign species while having minimal effects on other serum factors (22, 23). Both mouse and human serum samples were individually prepared in a solution containing 5% serum, 1% penicillin-streptomycin antibiotic cocktail, and 94% Dulbecco’s modified Eagle medium (DMEM) (Fig. 2). Aortic rings contained in 96-well plates were incubated in a 5% CO_2_, 37°C wet incubator for 48 h. Following the incubation period, aortas were immediately mounted on a pin myograph, and elastic modulus was assessed exactly as described in section above.

### Vascular Endothelial Function

To determine if the serum or culturing influences the magnitude of endothelial function with age, we sought to compare endothelium-dependent dilation (EDD) values from nonculture pressure myographs (111 P; DMT, Aarhus, Denmark), in which EDD is promptly assessed, and from the serum exposure (culture) pressure myographs (204CM; DMT), in which EDD is assessed after 24 h of serum perfusion (described in next section). Mice were euthanized via cardiac exsanguination under inhaled isoflurane anesthesia, and carotid arteries were immediately excised and mounted in a nonculture pressure myograph. Vasodilatory function was measured ex vivo via carotid artery EDD and endothelium-independent dilation (EID) in response to increasing doses of acetylcholine (ACh) and sodium nitroprusside (SNP), respectively, as described previously (10, 18). After vessels were preconstricted with phenylephrine (PE; 20 µM; Sigma-Aldrich, Cat. No. P6126), EDD was assessed by measuring increases in vessel diameter in response to increasing concentrations of ACh (1 × 10^−9^ to 1 × 10^−4^ M; Sigma-Aldrich, Cat. No. A6625). EID was assessed by measuring the increase in diameter in response to increasing concentrations of SNP, an exogenous NO donor (1 × 10^−9^ to 1 × 10^−3^ M; Sigma-Aldrich, Cat. No. 13755-38-9). Maximum diameter was determined from the greatest diameter observed from all the measurements. All dose responses are presented as percent dilations relative to maximum diameters to account for differences in baseline vessel diameters.

### Circulating Milieu-Related Vascular Endothelial Function

To assess the effects of the circulating milieu on endothelial function, mouse carotid artery tissue culturing, and subsequent endothelial function measurements were conducted using a culture pressure myograph system incorporating automated syringe drivers for intraluminal perfusion (Fig. 2). Before beginning other preparations, 1 L modified Krebs buffer, consisting of (in mM) 123 NaCl, 4.7 KCl, 1.2 CaCl_2_, 2.5 MgSO_4_, 16 NaHCO_3_, 0.026 EDTA, 1.18 KH_2_PO_4_, and 5.0 glucose, filtered through a ≤0.45-µm sterile membrane and stored at 4°C overnight, per pair of carotid arteries to be tested was brought to room temperature and adjusted to pH 7.2–7.25, which resulted in a bath pH close to 7.4 during incubation. Immediately following pH adjustment, a 5% CO_2_ gas mixture (21% O_2_-74% N_2_; Airgas) was bubbled into the buffer, which continued throughout the incubation process until the next day when testing began. Functionally, the reservoir bottle in which modified Krebs buffer is kept during incubation must have a means of delivering continuous gas flow throughout the solution, as well as drawing solution out from the bottom of the bottle and then a means of venting excess pressure from the top. The setup included a cap with three small openings: two with attached tubes allowing gas bubbling in through one and buffer flow out of the other and a third for pressure relief.

Before culturing, human serum samples were heat treated at 56°C for 30 min to inactivate the complement cascade. All serum samples (mouse and human) were individually prepared in a solution containing 5% serum, 1% penicillin-streptomycin antibiotic cocktail, and 94% modified Krebs buffer corrected to pH 7.3 (24, 25). The serum samples were sex matched to that of the donor animal and paired arteries from each mouse underwent intraluminal perfusion with serum from either condition (young or old serum). These dilute serum solutions were drawn into 10 mL syringes without needles, which were then attached to a Luer-Lok port on a tube connected to a microcannula on each myograph chamber. The tube and microcannula were then carefully filled with the serum solution in preparation for intraluminal perfusion (effort should be made to ensure no air bubbles are trapped in the syringe, line, or cannula during setup, as these could become trapped inside the carotid lumen during incubation and lead to tissue death). Syringes needed ≥10 mL of serum solution remaining when incubation began.

Immediately after euthanasia, common carotid arteries were excised from mice and each secured with nylon sutures to two glass microcannulas in a bath of physiological salt solution (PSS), containing (in mM) 144.9 NaCl, 4.7 KCl, 2 CaCl_2_, 1.17 MgSO_4_, 3.01 MOPS, 0.0255 EDTA, 1.2 NaH_2_PO_4_, 5.0 glucose, and 2.0 sodium pyruvate, set within a chamber apparatus. Arteries were cannulated such that the carotid bifurcation was at the vessel outlet and anterograde serum perfusion was attained. Each chamber was then attached to a heat controller set to 37°C. Syringes were affixed in pairs on syringe drivers which were set to a flow rate of 6–7 µL/min. Carotid arteries were pressurized to a constant 50–55 mmHg using a pneumatic pump with pressure lines each attached to a sealed waste collection bottle into which the serum solution would flow after passing through the lumen. This allowed directional flow of serum while maintaining constant pressurization throughout the incubation period (24, 25).

Once perfusion and pressurization were established, lines were connected directly to the baths to allow the superfusion of modified Krebs around the carotids for continuous nutrient replacement. A peristaltic pump system maintained a constant flow of ∼0.35 mL/min/chamber from the reservoir that was set up at the start of the session and then out of the chamber into a waste collection vessel. Crucially, the lines leading from the buffer reservoir, through the pump, and into the chambers needed to be narrow and as short as possible while still allowing the chambers to be arranged side by side without straining any components (in the case of this study, this distance was about 3 ft on average from reservoir to chamber with an internal diameter of 1/32 in.) and constructed of a relatively CO_2_-impermeable material like PVC to prevent loss of CO_2_ and subsequent excessive pH increase that could damage tissue and cause undesired precipitation of buffer constituents onto tissues and chamber surfaces.

Carotid arteries were incubated for 24 h under these conditions, at which point baths were replaced with PSS, and EDD and EID were tested using the methods described in section above. In contrast to the standard procedure, two EDD dose responses were measured consecutively with a 30-min recovery in between and values were averaged for each dose of ACh to account for any additional variability in the culture pressure myograph measurements.

### Statistics

Statistical analyses were conducted using GraphPad Prism version 10.1.0 (GraphPad Software; San Diego, CA). Standard versus cultured model comparisons were assessed using one-way ANOVA with statistical significance was set to α = 0.05 to detect the effects of serum exposure and age. Animal and participant characteristics and standard functional outcomes were assessed using unpaired *t* test with statistical significance was set to α = 0.05. Serum-exposure outcomes were assessed using paired *t* test with statistical significance was set to α = 0.05 given that different serum conditions were tested on arteries from the same mouse. Relations between specific variables in grouped data (young and ML/O serum exposure) were assessed using Spearman’s rank-based correlation. Data incorporated into these correlations were first assessed for normal multivariate distribution and residual normality (Henze–Zirkler normality test). Relations between specific variables in age-related subgroups were assessed using Pearson’s correlation. Data incorporated into these correlations were first assessed for normal distribution (Shapiro–Wilk normality test). Data are presented as means ± SE.

## RESULTS

### Animal Characteristics

Relative to young mice, old mice had higher aPWV (Table 1), suggesting that this cohort of animals had typical age-related arterial stiffening before euthanasia.

**Table 1.** Animal characteristics

	Young Mice	Old Mice
*n* (female/male)	15 (5/10)	18 (5/13)
Age, mo	3.8 ± 0.2	25.1 ± 0.1*
Aortic pulse wave velocity, cm/s	357 ± 13	402 ± 11*

Values are means ± SE; *n*, number of mice. *P < 0.05 vs. young mice.

### Model Comparison and Validation

#### Ex vivo exposure to serum from age-matched mice does not influence aortic stiffness.

Initially, we used the assessment of aortic elastic modulus as an approach to assess the effects of changes in serum on age-related aortic stiffening. We have previously established that aortic elastic modulus is higher in old versus young mice (*P* < 0.001) (18) (Table 2). To investigate the age-related effects of serum on aortic stiffness, we assessed elastic modulus in aortic rings following incubation with age-matched donor mouse serum. We observed age-related differences in elastic modulus in serum-exposed age-matched aortic rings (young aortic rings incubated with young serum vs. old aortic rings incubated with old serum, *P* = 0.002) (Table 2). Aortic rings from young mice incubated with serum from young donor mice had comparable elastic modulus values to aortic rings from young mice assessed immediately following excision (*P* = 0.911) (Table 2). Likewise, aortic rings from old mice incubated with old donor mice serum also had similar aortic elastic modulus to aortic rings from old mice assessed immediately following excision (*P* = 0.966) (Table 2).

**Table 2.** Model comparison using established methods (control) or following age-matched serum exposure

	Control		Serum Exposure	
	Young	Old	Young-Young	Old-Old
Aortic elastic modulus, kPa	4,508 ± 106	7,646 ± 536*	4,906 ± 575	7,386 ± 318*
Preconstriction, %	17 ± 2	16 ± 2	35 ± 2*	40 ± 2*,†
EDD EC_50_	−7.4 ± 0.5	−7.6 ± 0.8	−6.7 ± 0.5	−7.2 ± 0.5
Peak EDD, %	91 ± 3	71 ± 5*	92 ± 2	73 ± 5*
Peak EID, %	94 ± 1	93 ± 1	97 ± 1	97 ± 1

Values are means ± SE; *n* = 6–8 mice/group. EDD, endothelium-dependent dilation; EC_50_, half-maximal effective concentration; EID, endothelium-independent dilation. Statistics are one-way ANOVA. *P < 0.05 vs. young control. †*P* < 0.05 vs. old control.

#### Ex vivo perfusion of serum from age-matched mice does not influence endothelial function.

We next sought to compare the effects of the serum exposure assay to standard measures of endothelial function in the absence of serum exposure. Phenylephrine (PE)-induced preconstriction of the carotid arteries, a test of vessel viability and integrity (26), was higher in cultured vessels versus noncultured vessels in both young and old samples (*P* < 0.0001) (Table 2), suggesting that the cultured vessels were more sensitive to PE however both models were responsive and viable. Age-related differences in PE were not observed between nonculture vessels (*P* = 0.977), or in the serum cultured vessels (*P* = 0.257) (Table 2). Carotid artery sensitivity to ACh (EC_50_), an indication of basal artery health, was not different between age groups, as previously described (18), or between the standard versus serum exposure model (Table 2). In standard pressure myographs, common carotid arteries from old mice had ∼20% lower endothelial function compared with carotid arteries from young mice as assessed by peak EDD (Table 2). Using the serum perfusion model in a culture pressure myograph, we observed similar age-related differences in peak EDD following serum perfusion from age-matched animals (young arteries perfused with young serum vs. old arteries perfused with old serum, *P* = 0.006) (Table 2). Within age comparisons between the two experimental approaches, serum from young mice perfused through arteries from young mice demonstrated similar peak EDD to young mice arteries assessed immediately following excision (*P* = 0.998) (Table 2). Likewise, arteries from old mice had similar peak EDD between models (*P* = 0.999) (Table 2). We observed no differences among any groups in endothelium-independent dilation between any of the groups (Table 2), indicating that the age-related differences observed in endothelial function occurred in an endothelium-specific manner.

### Arterial Function following Exposure to Serum from Young and Old Mice

#### Serum from old mice stiffens young aortas.

**Figure 3.**
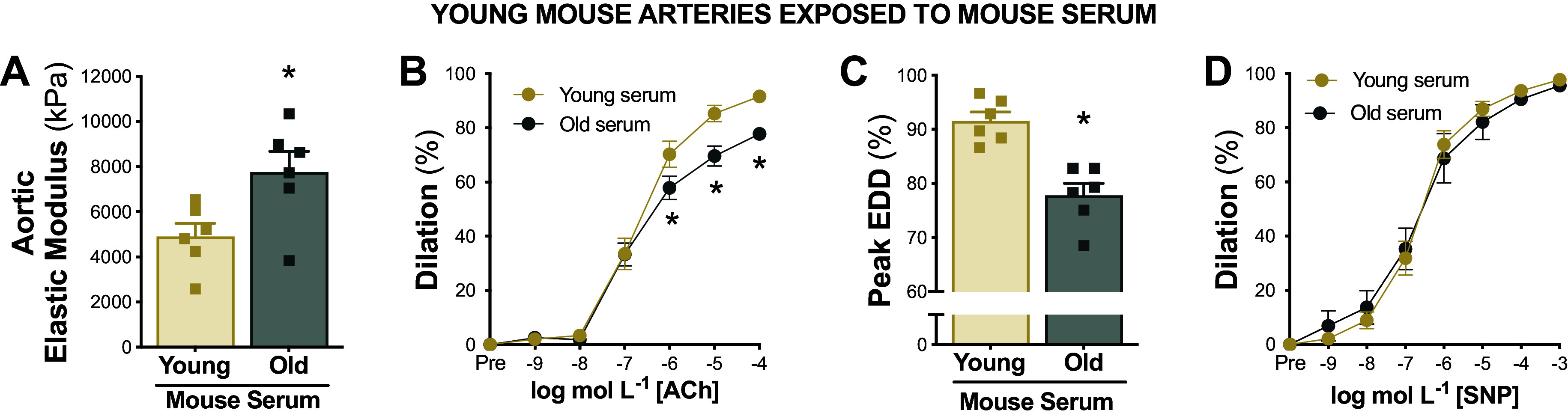
Serum from old mice induces arterial dysfunction in young arteries. Aortic elastic modulus (*A*), endothelium-dependent dilation (EDD) in response to increasing doses of acetylcholine (ACh; *B* and *C*), and endothelium-independent dilation in response to increasing doses of sodium nitroprusside (SNP; *D*) in young arteries following exposure to serum from young vs. old mice; *n* = 6/group. Data represent means ± SE. *P < 0.05 old vs. young mice serum exposure. Figure created with Photoshop.

To test if serum from old mice had similar effects on aortic stiffness as endothelial function, we exposed aortic rings from young mice to serum from young or old mice. Following serum incubations, young aortic rings exposed to old mice serum had higher elastic modulus compared with young aortic rings exposed to young mice serum (old serum, 7,762 ± 909 kPa vs. young serum, 4,906 ± 575 kPa, *P* = 0.003) (Fig. 3*A*).

#### Serum from old mice induces endothelial dysfunction in young arteries.

To assess if serum from old mice could induce endothelial dysfunction in young arteries, we exposed young mouse carotid arteries to serum from young or old mice. When compared with arteries perfused with serum from young mice, arteries perfused with serum from old mice had impaired EDD (peak EDD, young serum, 92 ± 2% vs. old serum, 78 ± 2%, *P* < 0.001) (Fig. 3, *B* and *C*). No significant differences were observed among groups in peak EID (*P* = 0.173) indicating that results were specific to the endothelium (Fig. 3*D*).

#### Serum from young mice reduces stiffness in old aortas.

**Figure 4.**
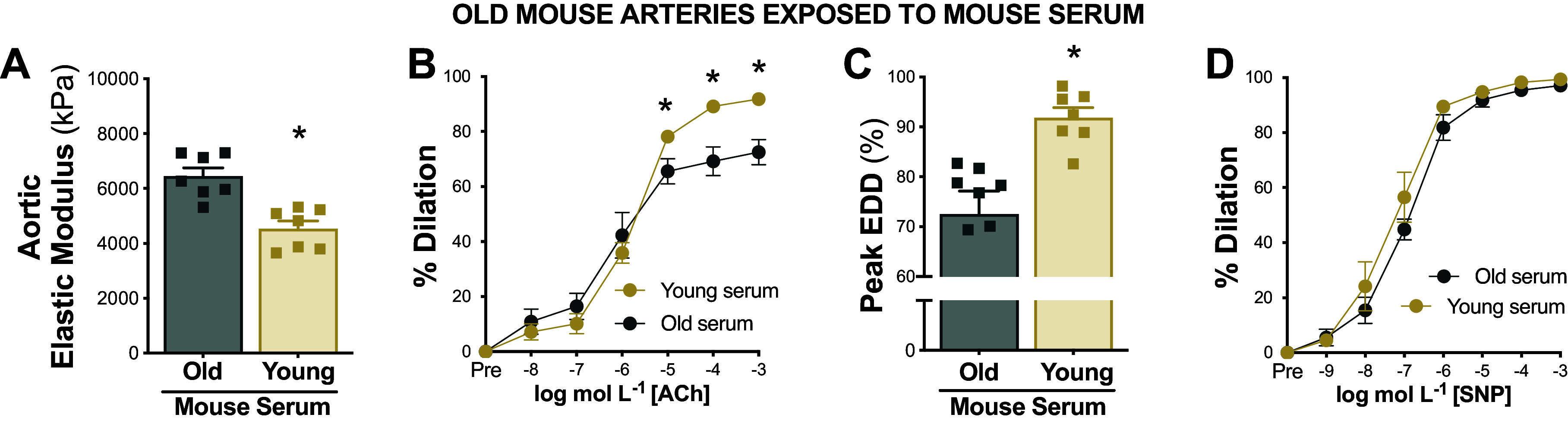
Serum from young mice improves arterial function in old arteries. Aortic elastic modulus (*A*), endothelium-dependent dilation (EDD) in response to increasing doses of acetylcholine (ACh) (*B* and *C*), and endothelium-independent dilation in response to increasing doses of sodium nitroprusside (SNP; *D*) in old arteries following exposure to serum from old vs. young mice; *n* = 7 or 8/group. Data represent means ± SE. *P < 0.05 old vs. young mice serum exposure. Figure created with Photoshop.

To test if serum from young mice had similar effects on aortic stiffness, we exposed aortic rings from old mice to serum from young or old mice and then measured aortic elastic modulus. Following serum incubations, old aortic rings exposed to young mice serum had lower elastic modulus compared with old aortic rings exposed to old mice serum (young serum, 4,540 ± 278 kPa vs. old serum, 6,452 ± 300 kPa, *P* = 0.009) (Fig. 4*A*).

#### Serum from young mice improves endothelial function in old arteries.

To determine if the circulating milieu from young animals could increase endothelial function in old arteries, we exposed arteries from old mice to serum from young or old mice and subsequently assessed EDD. When compared with old arteries perfused with serum from old mice, old arteries perfused with young mouse serum had greater endothelial function (peak EDD, old serum, 73 ± 5% vs. young serum, 92 ± 2%, *P* = 0.015) (Fig. 4, *B* and *C*). No significant differences were observed among groups in peak EID (*P* = 0.082) indicating that serum from young mice restored endothelial function in old arteries independent of changes in the vascular smooth muscle (Fig. 4*D*).

### Age-Related Arterial Function in Arteries Exposed to Human Serum

#### Participant characteristics.

Next, we aimed to translate our findings by performing similar experiments using serum from young and ML/O human adults. Young and ML/O participants had expected age-related differences in cfPWV, baFMD, and systolic and diastolic blood pressure (Table 3). There were no differences in body mass index, leisure time physical activity levels, or traditional CVD risk factors that are commonly assessed as circulating factors in serum (i.e., blood cholesterols, triglycerides, and glucose) between age groups. Differences in medications between groups are reported in Table 3.

**Table 3.** Participant characteristics

	Young Adults	Midlife/Older Adults
*n* (women/men)	10 (5/5)	10 (5/5)
Age, yr	23.5 ± 1.0	67.2 ± 2.9*
Brachial-artery flow-mediated dilation, %	9.3 ± 1.7	3.5 ± 0.5*
Carotid-femoral pulse wave velocity, m/s	5.1 ± 1.7	8.5 ± 0.3*
Systolic blood pressure, mmHg	112.6 ± 1.8	124 ± 2.9*
Diastolic blood pressure, mmHg	64.8 ± 1.5	73.5 ± 2.1*
Body mass index, kg/m^2^	23.2 ± 1.1	25.0 ± 1.0
Leisure time physical activity, MET h/wk	52.6 ± 9.6	42.6 ± 5.9
Total cholesterol, g/dL	158 ± 18	188 ± 15
HDL cholesterol, mg/dL	52 ± 3	58 ± 9
LDL cholesterol, mg/dL	98 ± 12	107 ± 11
Triglycerides, mg/dL	113 ± 13	113 ± 18
Fasting glucose, mg/dL	90 ± 3	92 ± 2
Medications		
β-Blocker	0/10	1/10
Angiotensin II-receptor blocker	0/10	1/10
Ca^2+^-channel blocker	0/10	2/10
Statin	0/10	2/10
Thyroid hormone	0/10	3/10
Antidepressant	0/10	4/10
Antihistamine	0/10	1/10
Hormonal contraceptives	3/10	0/10

Values are means ± SE; *n*, number of participants. MET, metabolic equivalent of task; HDL, high-density lipoprotein; LDL, low-density lipoprotein. *P < 0.05 vs. young adults.

#### Serum from ML/O adults stiffens young aortas.

**Figure 5.**
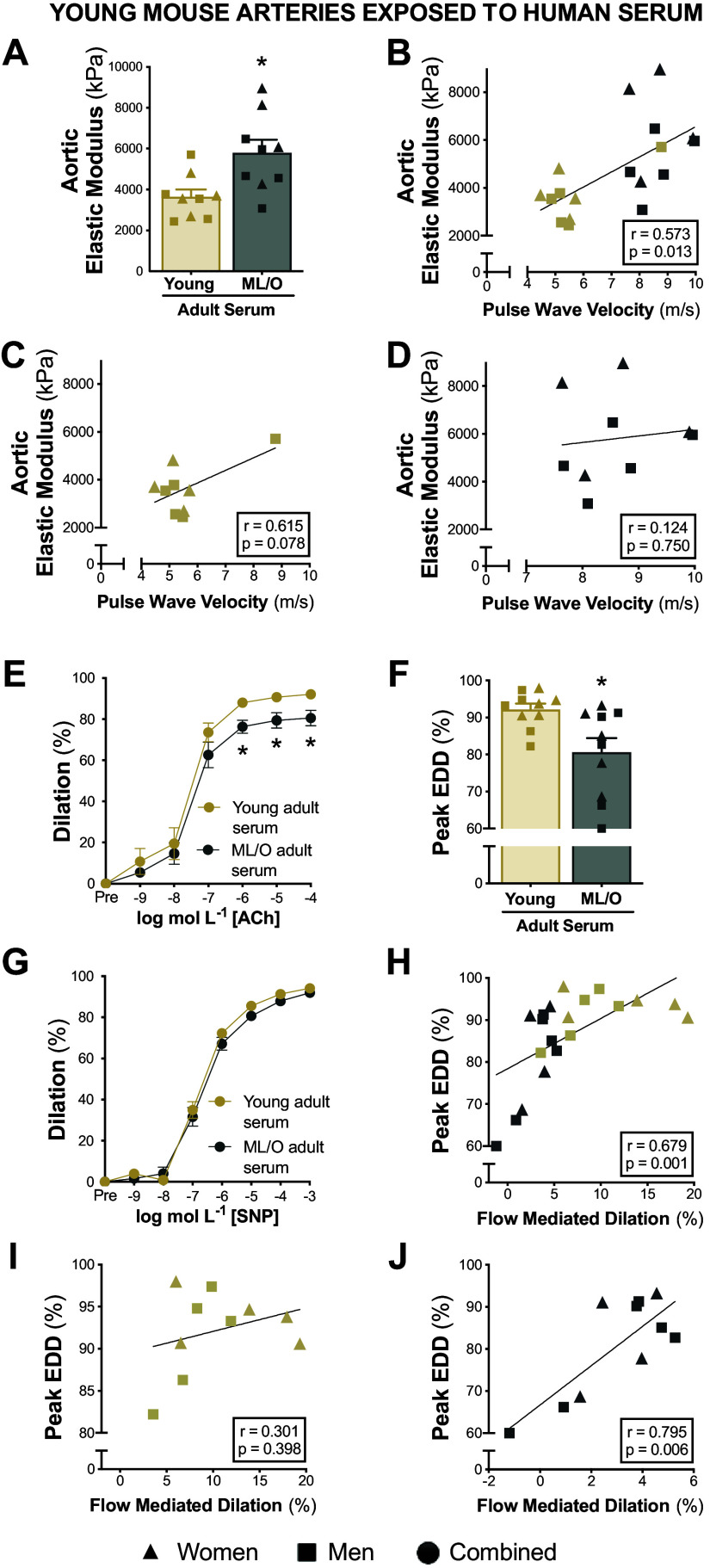
Midlife and older (ML/O) adult serum induces arterial dysfunction in young arteries. Elastic modulus in young aortas following exposure to young vs. ML/O adult serum (*A*). Pearson’s correlation between serum-induced differences in aortic elastic modulus and participant’s pulse wave velocity in all subjects (*B*), in the young adult subgroup (*C*), and in the ML/O adult subgroup (*D*). Endothelium-dependent dilation (EDD) in response to acetylcholine (ACh) (*E* and *F*) and endothelium-independent dilation in response to sodium nitroprusside (SNP; *G*) in young arteries following perfusion from young vs. ML/O adult serum. Pearson’s correlation between serum-induced differences in peak EDD and participants flow-mediated dilation in all subjects (*H*), in the young adult subgroup (*I*), and in the ML/O adult subgroup (*J*); *n* = 9 or 10/group. Data represent means ± SE. Triangles, women; squares, men; circles, combined sexes; gold shapes, young adults; gray shapes, ML/O adults. *P < 0.05 young vs. ML/O adult serum exposure. Figure created with Photoshop.

To test if serum from ML/O adults had similar effects on aortic stiffness, we exposed aortic rings from young mice to serum from young or ML/O adults and subsequently assessed elastic modulus. Aortic rings exposed to serum from ML/O adults had higher elastic modulus compared with aortic rings exposed to young adult serum (ML/O adult serum, 5,803 ± 630 kPa vs. young adult serum, 3,649 ± 357 kPa, *P* = 0.003) (Fig. 5*A*). To assess the contributions of the circulating milieu to in vivo aortic stiffness, we assessed the relation between serum-induced aortic stiffness to the participants’ aortic stiffness measurements. Aortic elastic modulus following serum exposure was related to the participants’ cfPWV values (*r* = 0.573, *P* = 0.013) (Fig. 5, *B*–*D*), suggesting that the circulating milieu likely influences (or contributes to) in vivo aortic stiffness of the donor.

#### Serum from ML/O adults impairs endothelial function in young arteries.

To test if serum from ML/O adults can induce dysfunction in young arteries, we exposed young mouse carotid arteries to serum from young or ML/O adults. When compared with young arteries perfused with serum from young adults, arteries perfused with serum from ML/O adults had impaired EDD (peak EDD, young adult serum, 92 ± 2% vs. ML/O adult serum, 81 ± 4%, *P* = 0.006), (Fig. 5, *E* and *F*). No differences in EID were observed among groups (*P* = 0.173) demonstrating that ML/O adult serum impaired EDD in an endothelium-specific manner (Fig. 5*G*). To determine if the circulating milieu may be a mechanism contributing to endothelial function, we assessed the relation of serum-induced endothelial function with the participant’s in vivo endothelial function. Interestingly, serum-induced differences in peak EDD were related to the participants’ baFMD values (*r* = 0.679, *P* = 0.001) (Fig. 5, *H*–*J*), indicating that the circulating milieu likely influences endothelial function in vivo.

#### Serum from young adults destiffens old arteries.

**Figure 6.**
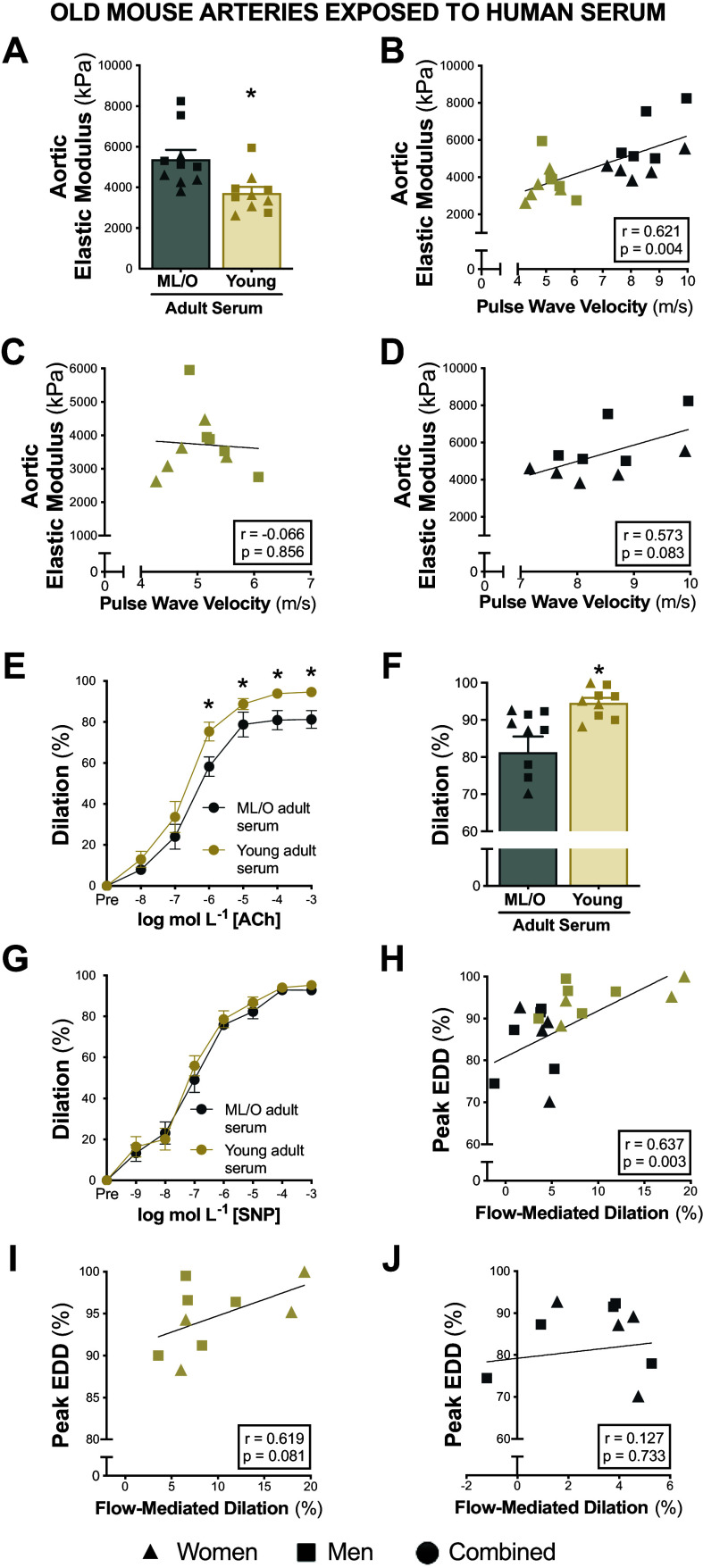
Young adult serum improves arterial function in old arteries. Elastic modulus in old aortas following exposure to midlife/older (ML/O) vs. young adult serum (*A*). Pearson’s correlation between serum-induced differences in aortic elastic modulus and participants’ pulse wave velocity in all subjects (*B*), in the young adult subgroup (*C*), and in the ML/O adult subgroup (*D*). Endothelium-dependent dilation (EDD) in response to acetylcholine (ACh; *E* and *F*) and endothelium-independent dilation in response to sodium nitroprusside (SNP; *G*) in old arteries following perfusion from ML/O vs. young adult serum. Pearson’s correlation between serum-induced differences in peak EDD and participant’s flow-mediated dilation in all subjects (*H*), in the young adult subgroup (*I*), and in the ML/O adult subgroup (*J*); *n* = 9 or 10/group. Data represent means ± SE. Triangles, women; squares, men; circles, combined sexes; gold shapes, young adults; gray shapes, ML/O adults. *P < 0.05 young vs. ML/O adult serum exposure. Figure created with Photoshop.

To test if serum from young adults had comparable effects on aortic stiffness, we exposed aortic rings from old mice to serum from young or ML/O adults and followed the measured elastic modulus. After serum incubations, aortic rings exposed to serum from young adults had lower elastic modulus compared with aortic rings exposed to serum from ML/O adults (young adult serum, 4,006 ± 341 kPa vs. ML/O adult serum, 5,414 ± 446 kPa, *P* < 0.001) (Fig. 6*A*). Aortic elastic modulus following serum exposure was related to the participants’ cfPWV values (*r* = 0.621, *P* = 0.004) (Fig. 6, *B*–*D*), further indicating that circulating milieu likely influences and contributes to in vivo aortic stiffness.

#### Serum from young adults improves endothelial function in old arteries.

To demonstrate that serum from young adults can increase endothelial function in old arteries, we exposed carotid arteries from old mice to serum from young or ML/O adults. When compared with old arteries perfused with serum from ML/O adults, arteries perfused with serum from young adults had enhanced endothelial function (peak EDD, ML/O adult serum, 81 ± 4% vs. young adult serum, 95 ± 1%, *P* = 0.015) (Fig. 6, *E* and *F*). No differences were observed among groups in EID (*P* = 0.438) indicating that the changes in endothelial function were specific to the endothelium (Fig. 6*G*). Serum-induced differences in peak EDD were related to the participants’ baFMD values (*r* = 0.637, *P* = 0.003) (Fig. 6, *H*–*J*), demonstrating again that the circulating milieu likely contributes to endothelial function in vivo.

## DISCUSSION

Elucidating the mechanisms by which aging impairs arterial function is essential for the identification of new therapeutic targets and effective therapies for the prevention and treatment of CVDs (27). Age-related changes in the circulating milieu may be a mechanism underlying arterial aging (7); however, conclusions regarding the direct role of the circulating milieu from mice and humans on arterial function are limited by current experimental methodologies.

In the present study, we established a novel ex vivo serum exposure model to test the causal impact of the circulating milieu on age-related arterial function. We first demonstrated that the serum exposure model accurately reflected age-related changes in endothelial function and aortic stiffness by comparing our findings to established ex vivo experimental techniques used to assess isolated artery function. These model comparisons demonstrate that age-related arterial function observed in vivo persists after age-matched serum exposure indicating that the serum exposure per se had limited effects on endothelial function and aortic stiffness.

Leveraging this serum exposure model, we then showed for the first time that the circulating milieu from young mice directly improves arterial function in old arteries and that the circulating milieu from old mice induces arterial dysfunction in young arteries. We next sought to extend the translatability of these findings to humans using serum samples from young and ML/O adults. Similar to the mouse circulating milieu, we found that exposure to serum from ML/O adults impairs function in young mouse arteries whereas the exposure to serum from young adults improves function in old mouse arteries. Collectively, these observations implicate the circulating milieu as an underlying mechanism of age-related arterial dysfunction. As such, our findings indicate that the circulating milieu is a potential therapeutic target for improving arterial function in ML/O adults.

### Impact of Circulating Milieu on Aortic Stiffness

Increases in large elastic artery stiffness with aging are a major independent predictor of age-associated clinical CVD (28), impaired glucose tolerance (29), kidney dysfunction (30), cognitive impairment (31), and diabetic retinopathy (32). We previously showed that the circulating milieu contributes to age-related aortic stiffening in mice via adverse changes in arterial redox signaling and remodeling of structural proteins in the arterial wall (10). Yet, limited studies exist exploring the implications of the young circulating milieu on treating/reversing aortic stiffness. Alterations in the circulating milieu with aging may induce aortic stiffening by reducing NO bioavailability, stimulating stress signaling pathways and inflammatory responses, and adverse arterial wall remodeling (33, 34). Circulating factors that are upregulated with aging such as oxidant radicals, proinflammatory cytokines, growth factors, matrix metalloproteins, and vascular adhesion molecules also contribute to age-related aortic stiffening (35). However, it is less clear which rejuvenating factors that are altered with chronological aging contribute to aortic stiffening.

In the present study, we found that the circulating milieu from mice and humans regulates age-related aortic intrinsic mechanical wall stiffness in a bidirectional manner. Using young and ML/O adult serum, we found consistent associations between the in vivo aortic stiffness of a human participant and the influence the serum exposure from the participant on the stiffness of the isolated mouse aorta. These results indicate that the circulating milieu is reflective of and implicated in aortic stiffening in vivo.

### Impact of Circulating Milieu on Vascular Endothelial Function

Vascular endothelial dysfunction is a key manifestation of age-related arterial dysfunction and a major antecedent to overt CVDs, including atherosclerosis and occlusive stroke (1). Endothelial cells make up the innermost layer of vascular tissues and are uniquely positioned to have direct contact with the circulating milieu (36). This suggests that endothelial cells may be more vulnerable to shifts in the circulating milieu due to their spatial proximity. Endothelial cells in culture exposed to plasma, serum, or components of the circulating milieu from old animals or ML/O adults show impaired endothelial cell function and promotion of age-related processes known to adversely affect endothelial cell health (17, 37–41).

A previous study using heterochronic parabiosis reported that sharing the circulating milieu of young mice improves aortic endothelial function in old mice (8). Mechanistically, the young circulating milieu may enhance endothelial function in old arteries by improving NO-mediated vasodilation, attenuating arterial oxidative stress, and favorably remodeling the vascular smooth muscle (8, 42). However, unlike our results, this prior work did not find impairments in arterial function in young mice exposed to the circulating milieu of old mice. This difference in findings may be due to the distinctive methodologies and experimental approaches. For instance, with in vivo approaches of heterochronic blood exchange such as heterochronic parabiosis, there are molecular (i.e., antioxidants) and cellular (i.e., immune and stress responses) defense mechanisms that may allow young mice to be resistant to the adverse effects of old mouse serum, ultimately precluding a clear interpretation of the results of the model. Furthermore, the heterochronic parabiosis model is a highly invasive experimental system in which several confounding factors exist (e.g., immune responses, shared circulations, and tissue oxygenation in conjoined animals) which may confound the interpretation of physiological outcomes (9).

In the current study, we isolated carotid arteries ex vivo and perfused them with serum on a pressure culture myograph to isolate the direct effects of the circulating milieu on endothelial function. This ex vivo approach is a promising tool to further explore how changes in the circulating milieu from interventions or cardiovascular pathologies contribute to improvements or impairments in endothelial function, respectively. Consistent with the aortic stiffness model, the serum perfusion endothelial function model could be used to elucidate the role of interventions, diseases, or individual compounds in the circulating milieu on endothelial function. Taken together, the model developed in this study provides fundamental evidence for the importance of the circulating milieu on age-related endothelial function and represents a powerful resource for future mechanistic studies.

### Experimental Considerations/Future Directions

Although traditional approaches of heterochronic blood exchange allow for in vivo investigation of the circulating milieu and may hold advantages for studying longer-term effects of the circulating milieu, the ex vivo serum exposure model used in this study has direct applications for further mechanistic studies on arterial function. Despite the short-term serum exposure, we observed profound and isolated changes in arterial function supporting previous findings of the physiological plasticity of arteries. Furthermore, we assessed for the first time the effects of the human circulating milieu on age-related aortic stiffening and endothelial function. We observed similar patterns in circulating milieu-related differences in arterial function between mouse and human serum exposure; however, there were some differences in the magnitude of difference between age groups. This may be due to the old mice being the human corollary age of ∼80 yr, whereas the ML/O adult samples used in this study were ∼67 yr with a larger age range, which may explain the smaller magnitude of difference in the human serum study. Thus, this experimental model may be used to compare the effects of serum on arterial function from diverse organisms, but age should be considered as an important confounding variable.

Future studies exploring components of the circulating milieu are required to determine the individual factors regulating circulating milieu-related changes in arterial function with aging by assessing blood fractions or using high-throughput omics-based approaches to identify putative targets. Furthermore, assessing molecular and structural serum-induced changes of vascular cells and the vascular wall may reveal the mechanisms by which the circulating milieu modulates arterial function. Extending these findings across the age continuum [i.e., adding a middle-aged group (30–50 yr)] will also elucidate further patterns in the effects of the circulating milieu on age-related arterial function.

### Conclusions

Arterial dysfunction plays a central role in CV-related morbidity and mortality in older adults (1); however, directly investigating mechanisms contributing to arterial aging in humans is challenging given the inaccessibility to arterial tissues. The serum exposure models developed in this study provide experimental insight into the causal impact of the circulating milieu in mediating adverse changes in arterial function with aging. Importantly, these experimental approaches also allow investigators to determine the role of the circulating milieu in mediating changes in arterial function *1*) with aging longitudinally over time in the same animals or human subjects and *2*) in response to lifestyle-based interventions and pharmacological treatments. As such, our findings have potential clinical significance for mitigating arterial dysfunction and its pathophysiological sequelae in humans. Preventing or reversing age-related changes in the circulating milieu toward that observed in young adults holds much promise for promoting CV health throughout the lifespan.

## DATA AVAILABILITY

Data will be made available upon reasonable request.

## GRANTS

This work was supported by National Institutes of Health Grants F31-HL165885 (to S.A.M.), K99/R00-HL151818 (to V.E.B.), R21-AG078408 (to D.R.S.), and K99-HL159241 (to Z.S.C.) and Horizon Europe Marie Sklodowska-Curie Actions Grant MSCA-IF-2019:892267 (to Y.B.Q.).

## DISCLOSURES

No conflicts of interest, financial or otherwise, are declared by the authors.

## AUTHOR CONTRIBUTIONS

S.A.M., N.T.G., M.J.R., V.E.B., Y.B.Q., D.R.S., and Z.S.C. conceived and designed research; S.A.M., N.S.V., N.T.G., R.V., and Y.B.Q. performed experiments; S.A.M. analyzed data; S.A.M., N.S.V., M.J.R., M.E.W., V.E.B., Y.B.Q., D.R.S., and Z.S.C. interpreted results of experiments; S.A.M. and N.S.V. prepared figures; S.A.M., N.S.V., and Z.S.C. drafted manuscript; S.A.M., N.S.V., N.T.G., R.V., M.J.R., M.E.W., V.E.B., Y.B.Q., D.R.S., and Z.S.C. edited and revised manuscript; S.A.M., N.S.V., N.T.G., R.V., M.J.R., M.E.W., V.E.B., Y.B.Q., D.R.S., and Z.S.C. approved final version of manuscript.
